# Case of Hydrophobia

**Published:** 1824-11

**Authors:** J. Robinson

**Affiliations:** Surgeon, 16th Lancers.


Art. VIII.—*Case of Hydrophobia.* By J. Robinson, Esq. Surgeon, 16th Lancers.

The subject of this case was twenty-three years of age, and newly arrived from Europe. He was bitten by a dog supposed to be mad, about seven o'clock in the evening of the 17th May; being in a state of intoxication at the time. Two fangs perforated the back of the right hand: one between the metacarpal bones of the ring and middle fingers; the other between the metacarpal bones of the middle and fore-finger.

Bleeding was promoted by warm water, and the potassa fusa freely applied. There was not much tumefaction or pain. The sloughs came away on the 4th June. He was purged, and put on the use of mercury: the system was soon affected; his throat became sore, and his general health a good deal impaired. The mercury was continued twenty-one days, but his mouth continued sore for many days after. On the 15th June, the wounds were healed; appetite and general health good; was discharged in good health on the 27th June.

It has been remarked, that he was intoxicated at the time he was bitten; nevertheless he evinced much concern for the consequences that might result from the accident. He was of a serious turn of mind, and one on whom such an accident was likely to make a strong impression.

He returned to the hospital, at six o'clock on the morning of the 18th of September, up to which period he had continued to do his duty. Said that he had felt very ill since the preceding evening, with a feeling of cramps in his stomach, bowels, and limbs; had drank to excess on the 16th, but did not attribute his illness to that circumstance; and said that he was quite sure it was the bite of the dog that was "coming against him." Pulse was frequent, tongue foul, skin moist, slight thirst, bowels confined. Was ordered a draught of infusion of senna and salts, which he could not be persuaded to take, and evinced a tendency to convulsions when it was presented to him: the same occurred frequently through the day. Had three dejections. Pulse fell to sixty-six, and soft; skin moist; tongue foul; said he had no thirst.

At nine P.M. symptoms were of a more decidedly hydrophobic character: the convulsive efforts were more frequent in their recurrence, and respiration was difficult and oppressed. Twenty-four ounces of blood were taken away, in hopes of giving temporary relief; but the convulsive paroxysms returned with increased severity, even during the operation of bleeding, and the breathing was not in the least relieved. An opiate draught was attempted to be given, but he could not be persuaded to swallow it; and he entreated to be let alone, as the bare mention of drinking, he said, would drive him into convulsions. He had no thirst; the paroxysms recurred with greater frequency and increased severity; and he expired, quite exhausted, after a very severe fit, a few minutes before five o'clock on the morning of the 19th September.

*Dissection.*—Some of the smaller arterial ramifications on the surface of the pia mater were beautifully injected; the vessels within the substance of the brain were also injected. The right lateral ventricle contained a very considerable quantity of fluid, perfectly limpid; the left contained much less; in the two there were probably three ounces. The minute ramifications upon the cerebellum were a good deal distended; as were the vessels on the pons varolii and upper part of the spinal marrow.

The sheath of the spinal marrow appeared unnaturally red along the dorsal vertebræ; and, on laying it open, the spinal marrow was observed to be unusually vascular. At the upper part of the cord, there were a number of minute vessels gorged with blood; but the lower presented more an inflammatory blush, the vessels not being so distinct, and the red tint more equally diffused. On the sheath being detached, there were observed several bits of a white substance adhering to the tunica arachnoidea, which possessed a considerable degree of firmness: they were generally of a circular or oval form, with serrated edges, as if the process of absorption had been going on.

The œsophagus and larynx were not inspected. The stomach showed some marks, but faint, of extensive inflammation; it was highest at the cardiac extremity. Many parts of the small intestines were slightly inflamed, particularly about the termination of the jejunum and middle of the ilium. The cœcum and right extremity of the colon were much distended with air; but the transverse and sigmoid arch, and rectum, were very much contracted.

